# How hand-made affects consumers’ perceived nutritional quality of organic food

**DOI:** 10.3389/fnut.2024.1448751

**Published:** 2024-11-07

**Authors:** Qin Liu, Jun Cao, Siyu Li, Yunyun Wei, Conghong Ma

**Affiliations:** ^1^Research Center for Industry Digitalization, Huainan Normal University, Huainan, Anhui Province, China; ^2^School of Administration, North Sichuan Medical College, Nanchong, Sichuan Province, China

**Keywords:** handmade, organic food, perceived nutritional quality, product quality, handicraft cultural identity

## Abstract

This study analyzed the impact of handmade in depth on consumers’ perceived nutritional quality of organic food through three experiments. Experiment 1 found that handmade significantly enhanced consumers’ positive perceptions of nutritional quality of organic food. Experiment 2 revealed the mediating role of handicraft cultural identity between handmade and perceived nutritional quality. Experiment 3 further explored the moderating role of perceived authenticity on the impact of handmade. We found that these factors above significantly enhanced the positive effect of handmade on perceived nutritional quality. The findings suggest that handmade directly affected consumers’ perceived nutritional quality of organic food, which could be further strengthened through the mediating effect of handcraft cultural identity and the moderating effect of perceived authenticity. These findings provide important guidance for organic food manufacturers and marketers on marketing strategy-making that can help better meet consumer demand for high-quality organic food.

## 1 Introduction

Handmade refers to the production and manufacture of products by hand with raw materials and tools. In addition to its unique role in the making experience (1), product novelty (2), and product attractiveness (3), handmade is crucial in enhancing food taste and quality. In the past, many bodies have invested significant resources in defining and researching techniques and processes, to improve the quality and taste of handmade food and enhance consumers’ purchasing intentions (4). In recent years, although organic food has received consumers’ appreciation, the organic food production has gradually shifted from handmade to machine-made. It has been shown that the production process of organic food is important for consumer product perception (5). However, it is reasonable to question whether the different production processes of organic food are really effective in influencing consumers’ perceptions of organic food nutritional quality. More importantly, there is a lack of evidence in the existing research in marketing to address relevant issue.

Perceived nutritional quality of organic food shows how consumers perceive and evaluate the nutritional quality of organic food (6). There is a large amount of research on organic food quality perception, and it is becoming a trend in the marketing field (7–9). Previous studies have shown that the nutritional quality of organic food is affected by the processing technology involved in food production (10, 11). Perito et al. (12) suggested that the processing technology decreases the natural nutrient quality of organic food, and Sridhar et al. (13) again demonstrated that the extraction technology affects the food quality. It is evident that there is still some room for exploration and research on how handmade affects food quality. A recent study has shown that the nutritional quality of handmade food is better than machine-made ones (14). Although the above studies have discussed the advantages of handmade, they have not further discussed the effects of handmade on consumers’ perceived nutritional quality of organic food, as well as their theoretical and practical values have not been recognized. More importantly, the mechanisms and effects of handmade on consumers’ perceived nutritional quality of organic food are not yet clearly defined. Therefore, this study tried to answer the following questions: does handmade positively affect consumers’ perceived nutritional quality of organic food? What is the mechanism by which handmade affects consumers’ perceived nutritional quality of organic food?

To fill the above research gap, this study developed a framework to explore whether and how handmade positively affects the perceived nutritional quality of organic food. *This study hypothesized that handmade organic food can evoke consumers’ perceptions of food quality, which is associated with the potential consumers, regarding to the food processing methods used in handcrafting.* Although handmade has received extensive attention and been widely analyzed (15, 16), few studies have discussed the relationship between handmade and the perceived nutritional quality of organic food. Based on this, first, we proposed that the handmade production of organic food can increase their nutritional value, which in turn contributes to consumers’ perceived nutritional value. Second, from the perspective of product culture, we discussed the mediating role of handicraft cultural identity on the relationship between handmade and perceived nutritional value of organic food. Third, we focused on the moderating role of consumers’ perceived authenticity, and explored in depth the interaction effect between perceived authenticity and handmade on consumers’ perceived nutritional quality of organic food.

This study made the following important contributions to handmade and perceived nutritional quality of organic food. First, although studies on handmade are relatively common, few studies have focused on its functions and impacts on food nutritional quality. Therefore, this study contributed to enriching the existing literature on handmade and perceived nutritional quality of organic food. Handmade and perceived nutritional quality of organic foods have been combined, to discuss the relationship and influencing mechanisms between the two. Second, this study explored the mediating role of handicraft cultural identity and revealed its importance in enhancing the perceived nutritional quality of organic food. Third, this study identified the moderating role of perceived authenticity, suggesting that consumers tend to purchase handmade organic food with higher authenticity.

## 2 Theoretical framework and research overview

The impact of processing methods on food quality is multifaceted, which directly affect food safety, nutritional value, taste, and shelf life (17). Various processing technologies, such as thermal treatment, freezing, drying, and microwave heating, can influence the nutritional components of food in some extent (18). For instance, high-temperature processing may lead to the loss of certain nutrients, particularly heat-sensitive vitamins and some bioactive substances. Meanwhile, factors such as temperature, humidity, and time during the processing can also affect the nutritional content and taste of food (19). Moreover, innovations in food processing technology have brought new opportunities for enhancing food quality. The application of high-pressure pasteurization technology, for example, can pasteurize at lower temperatures, which effectively keep the nutritional components and taste of food. Studies have found that traditional processing methods, primarily based on craftsmanship (20, 21), are more likely to generate cultural identification among consumers, while machine-made in factories could be perceived as mass food production, thereby influencing consumers’ perceptions on food quality.

Handmade refers to the processing with the handwork and skills of craftsmen, rather than relying exclusively on mechanized production (22). This type of production emphasizes personalization, customization and fine craftsmanship (23). Despite the advantages in efficiency and cost of the modernized production methods, handmade still has an irreplaceable value (24). It is not only a means of production, but also a manifestation of cultural expression and individual creativity (25). In the context of globalization and industrialization, handmade offers an alternative to mass production and satisfy consumers’ quest for uniqueness, individuality, and cultural value (26). The concept of handmade means far beyond the physical process itself, encompassing aspects such as personalization, cultural heritage, skills demonstration, customized services, emotional value, environmental friendliness, economic contribution, and educational significance (27). In modern society, handmade continues to play an important role as a cultural and economic phenomenon, which is closely attached to consumers’ needs and market trends (28). Compared with machine-made food, handmade food pays more attention to the raw material selection and details of the processing, thus it is more favorable in nutritional value and taste (29). In this case, people will have higher perceived nutritional value of handmade food.

*On the one hand, individuals engaged in the processing of handmade food tend to place greater emphasis on the selection of raw materials and the complexity of the processing details* (30). *In the case of handmade, the selection of ingredients and the processing are controlled by human hands, so that more attention can be paid to their quality and freshness* (31)*. Some scholars posited that, compared to machine-made food, those handmade food are perceived as healthier and more nutritious* (32)*. However, this is not an absolute rule, as machine-made food can also be rich in nutritional value. Nevertheless, research indicated that consumers are more inclined to purchase foods that are based on handmade methods, as they can feel a greater sense of “food naturalness” from these products* (20). On the other hand, handmade food focuses more on the quality and mouthfeel of the food (33). In the process of handmade, craftsmen can adjust with their tastes, which can better satisfy people’s needs for food. Compared to machine-made food, handmade food is more responsive to people’s tastes and needs, values the quality and texture of the food, and therefore is more palatable to people.

Handmade food, as an integral component of traditional dietary culture, also plays a crucial role in modern society. Although industrialization and commercialization have altered people’s dietary habits, research indicated that handmade food still holds an important position in food culture (31). This mode of production not only satisfies people’s pursuit of gourmet experiences, but also reflects the inheritance and promotion of traditional culture. Handmade food serves as a bridge between the past and the present, tradition and modernity, allowing individuals to feel the depth and warmth of culture while enjoying culinary delights (34). Therefore, we should pay more attention to and support handmade food to consistently play its irreplaceable role in contemporary society (35).

Cultural identity refers to the sense of belonging that individuals or groups have toward their culture, which could stem from their shared values, beliefs, customs, language, and other cultural characteristics (36, 37). In the context of handmade food, cultural identity specifically denotes consumers’ appreciation for the traditions and craftsmanship of hand-making, as well as the historical and cultural value embodied by handmade (38). The concept of handcraft cultural identity involves consumers’ admiration for the skills, creativity, and adherence to traditional methods of artisans. This sense of identity not only strengthens consumers’ emotional connection to the food but also enhances their perception of the food quality and nutritional value (39). Consumers are likely to perceive handmade foods as more natural and healthy, and of greater cultural worth, as these products reflect higher human involvement and attention to details compared to mass mechanized production (40).

When consumers have a profound identification with the cultural value of handmade food, they are more likely to believe that these foods possess higher quality (41). This belief arises because handcraft has been seen as the continuation of traditional craftsmanship, which, compared to modern industrial production, is perceived to better preserve the natural attributes and nutritional value of food (42). Furthermore, the cultural identity with handmade food may lead to more positive emotional responses from consumers, thereby influencing their perception and evaluation of food nutritional quality (31).

Perceived authenticity refers to people’s perceptions and judgments as to the information authenticity (43). In the food domain, the moderation of perceived authenticity has a significant impact on people’s perceived nutritional food quality (44). Handmade food requires people to be hands-on during the production process, which makes the process of food preparation more intimate and tactile. In the process, people need to choose and mix ingredients, as well as control the heat, which all require certain skills and experience. Therefore, food handmade is not only a way of production, but also an inheritance of skills and culture (45). In this process, people can feel the charm of traditional culture, and the cultural characteristics of different regions and nationalities, through the taste and texture of food.

Therefore, this study argued that perceived authenticity moderates the relationship between handmade food and perceived nutritional quality. For better perceive the nutritional value in handmade food, people need to understand the nutritional value of ingredients, the technologies and methods used in the preparation, as well as the methods of food preservation and consumption (46). At the same time, people also need to recognize and perceive the authenticity of the food nutritional value (47). In modern society, we should pay more attention to handmade food and make it part of our food culture, as well as make our cuisine healthier and more delicious.

Based on above analysis, the following hypotheses were proposed.

H1: Handmade (vs. machine-made) in food production is more able to enhance consumers' perceived nutritional quality of organic food.

H2: Handcraft cultural identity mediates the relationship between handmade (vs. machine-made) and perceived nutritional quality of organic food.

H3: Perceived authenticity moderates the relationship between handmade (vs. machine-made) food and perceived nutritional quality.

**Figure 1:**
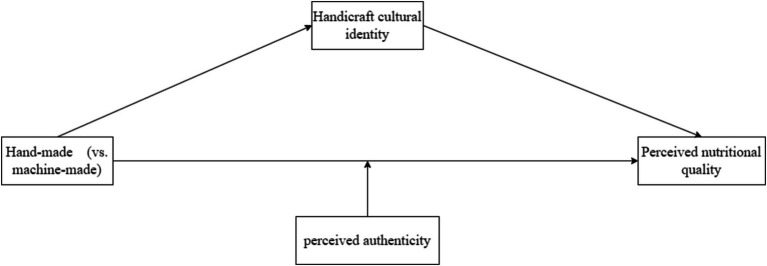
Theoretical model diagram.

The conceptual model is shown in Figure 1.

## 3 Overview of the study

This study explored the impact of handmade on consumers’ perceived nutritional quality of organic food, as well as its mechanisms and boundary conditions, through three experiments. Experiment 1 verified the main effect of handmade (vs. machine-made) on perceived nutritional quality (H1). Experiment 2 investigated the mediating role of handicraft cultural identity (H2). Experiment 3 analyzed the moderating role of perceived authenticity (H3). The participants in the experiments were randomly divided into two groups, one group was told that the food was handmade and the others were machine-made. The handmade group emphasized the combination of traditional craftsmanship and modern design, while the machine group highlighted the precision and efficiency of modern technology. Below are the criteria for recruiting participants in our study:(1) Have made at least three purchases of organic food products within the past 2 months. (2) Regularly follow information related to organic food. (3) Have a particular preference for handmade items. (4) Are able to recognize and understand the organic food logo. We manipulated the concept of handmade by describing the stimulus pictures in detail. As shown in Table 1. Demographic information for three experiments is shown in Table 2. Three experimental variables and their measurement questions, are shown in Table 3.

**Table 1:** Research framework table.

Study	Study1	Study2	Study3
Purpose	To test for main effect (H1)	To test the mediating effect of handicraft cultural identity (H2)	To test the moderating effect of perceived authenticity (H3)
Independent variable	Hand-made (vs. machine-made)	Hand-made (vs. machine-made)	Hand-made (vs. machine-made)
Dependent variable	Perceived nutritional quality of organic food	Perceived nutritional quality of organic food	Perceived nutritional quality of organic food
Mediators	-	Handicraft cultural identity	-
Moderator	-	-	Perceived authenticity
Methods	ANOVA	ANOVA	ANOVA
		PROCESS 4	PROCESS 1
Stimulus picture	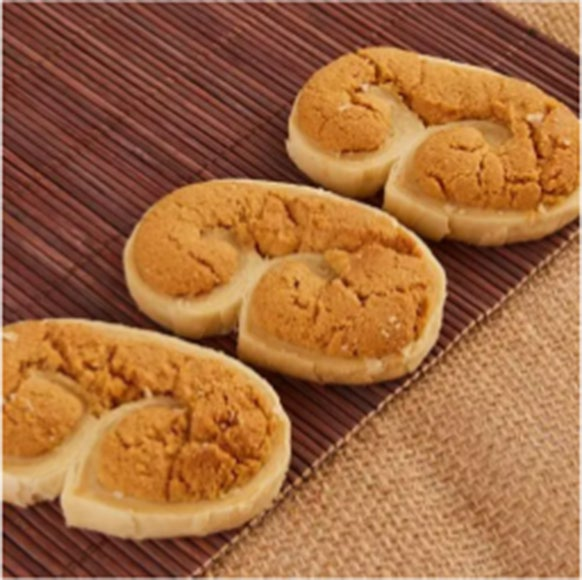	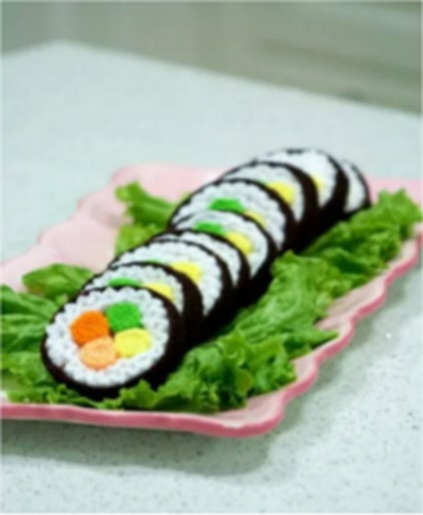	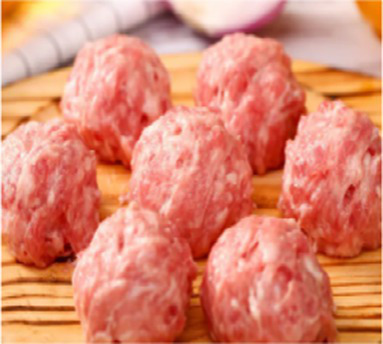
Product description	The organic food in the picture above is made of organic raw materials such as organic flour, organic butter, organic granulated sugar and organic dried peach. The materials are clean and pollution-free, and the environment is hygienic to ensure food safety.	The products in the picture above are made from organic raw materials such as organic rice, organic vegetables, and organic seafood. Through cooking, sushi-wrapping and other processes, the use of natural pollution-free organic ingredients contributes to healthier and safer products.	The organic food in the picture above is made of organic meat, organic seasonings and accessories. All materials are made of organic raw materials to reduce pollution and ensure the health of the product.
Results	Supported H1	Supported H2	Supported H3

**Table 2:** Demographic information for three experiments.

Variable	Items	Experiment 1		Experiment 2		Experiment 3	
		Frequency	Proportion	Frequency	Proportion	Frequency	Proportion
Gender	Male	141	47.2%	139	51.50%	143	51.10%
	Female	158	52.8%	131	48.50%	137	48.90%
Age	18–25 years old	74	24.7%	67	24.80%	78	27.90%
	26–40 years old	92	30.80%	66	24.40%	82	29.30%
	41–60 years old	66	22.1%	63	23.30%	53	18.90%
	Over 61 years old	67	22.40%	74	27.40%	67	23.90%
Education background	Primary school	49	16.40%	47	17.40%	47	16.80%
	Junior high school	48	16.10%	50	18.50%	45	16.10%
	Technical secondary school, high school	47	15.70%	41	15.20%	41	14.60%
	Junior college	67	22.40%	60	22.20%	64	22.90%
	Undergraduate college	64	21.40%	56	20.70%	63	22.50%
	Postgraduate	24	8.00%	16	5.90%	20	7.10%

**Table 3:** Three experimental variables and their measurement questions.

Variable	Definition	Measurement problem
Handmade	Handmade refers to the process of making objects through the handwork and skills of craftsmen	
Perceived nutritional quality of organic food	Perceived nutritional quality of organic food shows how consumers perceive and evaluate the nutritional quality of organic food	After you learnt about the process of making the organic food mentioned above, do you agree that the organic food is rich in nutrients?
Handicraft cultural identity	Handmade crafting cultural identity refers to the psychological process by which individuals or groups express affirmation and a sense of belonging to the cultural values and traditional craftsmanship of their own nation or community through the activity of handmade arts.	Do you agree that you are ready to learn about its history, traditions, and customs through practice?
		After seeing the above organic foods, do you agree that you have a strong sense of belonging and attachment to your own ethnic group?
		After seeing the organic foods mentioned above, do you agree that there are things you often do to better understand your ethnic background?
Perceived authenticity	Perceived authenticity refers to people’s perceptions and judgments as to the veracity of information	Do you agree or disagree that the organic food in the above picture is real?
		Do you agree that the organic food in the picture above is made with authentic traditional techniques?

## 4 Experiment 1: main effect of handmade-organic foods perceived nutritional quality

### 4.1 Experimental design

Experiment 1 aimed to explore the main effect of the handmade on the perceived nutritional quality of organic food. We designed a one-factor between-subjects ANOVA (handmade vs. machine-made) and 299 participants were recruited on Credamo.^1^ Among them 141 (47.2%) were male and 158 (52.8%) were female. The age distribution of all the participants was 24.7% aged 18–25 years, 30.8% aged 26–40 years, 22.1% aged 41–60 years, and 22.4% aged 61 years and above. There were 149 participants in the handmade group and 150 participants in the machine-made group.

The same picture of an organic food was shown to two groups of participants, but were presented with different information about how it was made. We assigned a value of 1 to the handmade group and a value of 2 to the machine-made group, and then asked the participants, “After you learnt about the process of making the organic food mentioned above, do you agree that the organic food is rich in nutrients?” (1 = strongly disagree, 7 = strongly agree), which was used to measure the participants’ perceived nutritional quality of the organic food. Last, we collected demographic information of the participants (Cronbach’s *α* = 0.678).

### 4.2 Experimental results

There was a main effects test. We conducted a one-way ANOVA with handmade as the independent variable and perceived nutritional quality of organic food as the dependent variable. The experimental results showed that consumers’ perceived nutritional quality of organic food in the handmade group (M = 5.83, SD = 1.584) was significantly higher than that in the machine-made group (M = 5.1, SD = 1.717), *F* (1, 297) = 14.409, *p* < 0.001. H1 was verified.

There was a control variable analysis. Given the study of Bärebring et al. (48), it was found that gender is a significant influence on consumers’ perceived nutritional quality of organic food. Therefore, in this study, a one-way ANOVA was conducted with gender as the independent variable and perceived nutritional quality of organic food as the dependent variable. The experimental results showed that gender does not have a significant effect on consumers’ perceived nutritional quality of organic food [*F* (1, 297) = 0.044, *p* = 0.833]. Therefore, consumers’ gender would not significantly affect the experimental results, again validating H1.

### 4.3 Discussion

Experiment 1 demonstrated that handmade has a significant effect on the perceived nutritional quality of organic food. Specifically, consumers perceived higher nutritional quality of handmade organic food. At the same time, we analyzed that gender as a control variable had no significant effect on the perceived nutritional quality of organic food, enhancing the reliability of the experiment. Despite the above findings in Experiment 1, Experiment 1 did not further discuss the internal mechanisms and boundary conditions between handmade and perceived nutritional quality of organic food. Therefore, we introduced handicraft cultural identity as a mediating variable in Experiment 2, proposing that handicraft cultural identity mediated the relationship between handmade and perceived nutritional quality of organic food.

## 5 Experiment 2: the mediating role of handicraft cultural identity

### 5.1 Experimental design

Experiment 2 aimed to explore that handicraft cultural identity mediates the relationship between handmade and perceived nutritional quality of organic food. We designed a one-factor between-subjects ANOVA (handmade vs. machine-made) and recruited 270 participants on Credamo (see text footnote 1). Among them, 139 (51.5%) participants were male and 131 (48.5%) participants were female. The age distribution of all the participants was 24.8% aged 18–25 years, 24.4% aged 26–40 years, 23.3% aged 41–60 years, and 27.4% aged 61 years and above. There were 130 participants in the handmade group and 140 participants in the machine-made group.

We showed the same food picture of organic sushi to both groups of participants, but used different content to describe how it was made. We assigned the handmade group a value of 1 and the machine-made group a value of 2. We then measured participants’ handicraft cultural identity with a scale adapted from Phinney and Ong (49), as “Do you agree that you are ready to learn about its history, traditions, and customs through practice?” (1 = strongly disagree, 7 = strongly agree). Along with this, we measured the participants’ perceived nutritional quality of organic food.

Based on Wilcox et al. (50), consumers’ product preferences may influence consumers’ perceived nutritional quality of organic foods. Therefore, we took consumer product preference as a control variable. Participants were asked “You need to buy organic food, do you agree that you would choose the organic food as shown in the figure above?” (1 = strongly disagree, 7 = strongly agree) (50). Last, we collected demographic information of the participants. (Cronbach’s *α* =0.736).

### 5.2 Experimental results

There was a main effects test. We applied one-way ANOVA with handmade as the independent variable and perceived nutritional quality of organic food as the dependent variable. The results of the experiment showed that consumers’ perceived nutritional quality of organic food in handmade group (M = 5.58, SD = 1.674) was significantly higher than that in the machine-made group (M = 4.94; SD = 1.901), *F* (1, 268) = 8.804, *p* = 0.003. H1 was verified.

**Figure 2:**
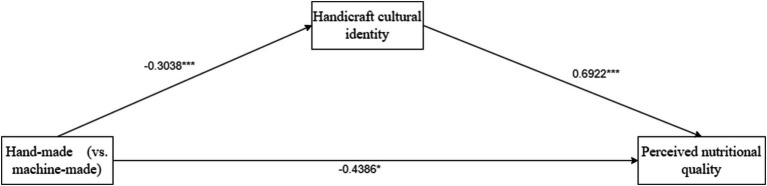
The mediating role of handmade cultural identity in Experiment 2.

There was a mediating effect analysis. We took handmade as the independent variable, perceived nutritional quality of organic food as the dependent variable, and handicraft cultural identity as the mediating variable, and used Process Model 4 to analyze the mediating role of handicraft cultural identity on the relationship between handmade and perceived nutritional quality of organic food (Bootstrap sample: 5,000; Igartua and Hayes, 63). The experimental results showed that the coefficient of handmade-handicraft cultural identity was −0.3038*; the coefficient of handmade-perceived nutritional quality of organic food was −0.4386*; and the coefficient of handicraft cultural identity-perceived nutritional quality of organic food was 0.6922***. Overall, the mediation of handmade-handicraft cultural identity-perceived nutritional quality of organic food was significant (*β* = −0.2103, SE = 0.0904, 95% CI = [−0.4028 to −0.048]). Therefore, handicraft cultural identity completely mediated the relationship between handmade and perceived nutritional quality of organic food. H2 was verified. As shown in Figure 2.

There was a control variable analysis. Considering that product preference may influence consumers’ perceived nutritional quality of organic food, we again analyzed the impact of handmade on perceived nutritional quality of organic food by taking product preference as a covariate. The experimental results showed that product preference did not have a significant impact on perceived nutritional quality of organic food [*F* (1, 268) = 6.23, *p* = 0.013]. Therefore, product preference would not significantly affect the experimental results. H2 was verified.

### 5.3 Discussion

Experiment 2 demonstrated the mediating role of handicraft cultural identity, and we found that consumers’ handicraft cultural identity effectively mediated the relationship between handmade and perceived nutritional quality of organic food, which better elaborated the internal mechanism between the two. Experiment 2 also verified that consumers’ product preferences had no significant effect on the experimental results. Despite the above findings, Experiment 2 did not discuss whether there is a moderating role between handmade and perceived nutritional quality of organic food. To fill in the gaps of the above experiments, we introduced perceived authenticity as a moderating variable in Experiment 3 and tried to explore its moderating effect on the relationship between handmade and perceived nutritional quality of organic food.

## 6 Experiment 3: the moderating role of perceived authenticity

### 6.1 Experimental design

Experiment 3 aimed to investigate the moderating effect of perceived authenticity on the relationship between handmade and perceived nutritional quality of organic food. We designed a 2 (handmade vs. machine-made) × 2 (perceived authenticity: high vs. low) ANOVA and 280 participants were recruited on Credamo (see text footnote 1). Among them 143 (51.1%) participants were male and 137 (48.9%) participants were female. The age distribution of all the participants was 27.9% aged 18–25 years, 29.3% aged 26–40 years, 18.9% aged 41–60 years, and 23.9% aged 61 years and above. There were 142 participants in the handmade group and 138 participants in the machine-made group.

We showed the same food picture of organic meatballs to both groups of participants, but used different content in describing how they were made. We assigned the handmade group a value of 1 and the machine-made group a value of 2. We then measured the participants’ perceived authenticity with a scale adapted from Wilcox et al. (50), as “Do you agree that the organic food in the picture is real?” (1 = strongly disagree, 7 = strongly agree). Along with this, we measured the participants’ perceived nutritional quality of the organic food. Last, we collected demographic information of the participants. (Cronbach’s *α* = 0.651).

### 6.2 Experimental results

There was a main effects test. We employed one-way ANOVA with handmade as the independent variable and perceived nutritional quality of organic food as the dependent variable. The results of the experiment showed that: M_handmade_ = 6.01, SD_handmade_ = 1.376, M_machine-made_ = 4.67; SD_machine-made_ = 1.4, *F* (1, 278) = 65.256, *p* < 0.001. It can be seen that consumers’ perceived nutrient quality of organic food is significantly higher in handmade group than that in machine-made group. H1 was verified.

There was a moderating effect analysis. We analyzed the moderating role of perceived authenticity on the relationship between handmade and perceived nutritional quality of organic food, taking handmade as the independent variable, perceived nutritional quality of organic food as the dependent variable, and perceived authenticity as the mediating variable, with Process Model 1 (Bootstrap sample: 5,000; Igartua and Hayes, 63). The experimental results showed that handmade had a significantly negative effect on perceived nutritional quality of organic food (*β* = −1.2297, *p* < 0.001); perceived authenticity had a significantly positive effect on perceived nutritional quality of organic food (β = 0.3054, *p* < 0.001); and the interaction effect between perceived authenticity and handmade had a significantly negative effect on consumers’ perceived nutritional quality of organic food (β = −0.2872, SE = 0.1448, *p* = 0.048, 95% CI = [−0.5723 ~ −0.0022]). It can be seen that perceived authenticity effectively moderated the relationship between handmade and consumers’ perceived nutritional quality of organic food. H3 was verified.

**Figure 3:**
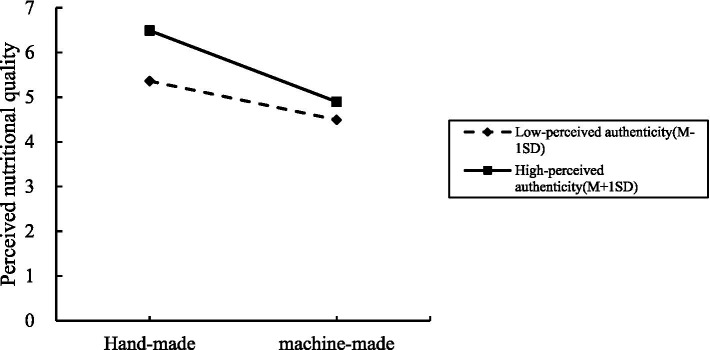
The moderating effect of perceived authenticity in Experiment 3.

As shown in Figure 3.

### 6.3 Discussion

Experiment 3 verified the moderating effect of perceived authenticity on the relationship between handmade and perceived nutritional quality of organic food. Consumers with high handicraft cultural identity perceived higher nutritional quality of organic food, and those with low handicraft cultural identity perceived lower nutritional quality of organic food. Consumers perceived higher nutritional quality of handmade organic food compared to machine-made organic food.

## 7 General discussion

This study discussed how organic foods processed through handmade and machine-made influence consumers’ perceptions of nutritional quality. In this research, organic food is defined as food produced in accordance with organic agriculture standards, which prohibit the use of chemically synthesized pesticides, fertilizers, growth regulators, feed additives, and genetic engineering techniques (51). Handmade food refers to food made through manual skills and traditional methods, typically produced by individual artisans or small-scale manufacturers with no dependence on mechanized production processes (52). This study utilized random sampling in mainland China and recruited participants through Credamo (see text footnote 1), an online platform. The researchers conducted three experiments to explore the impact of handmade on consumers’ perceived nutritional quality of organic food, as well as its underlying mechanisms and boundary conditions. Experiment 1 found that handmade enhances consumers’ perception of the nutritional quality of organic food (H1). Experiment 2 indicated that handicraft cultural identity mediates the impact of handmade on perceived nutritional quality (H2). Experiment 3 discovered that perceived authenticity moderates the relationship between handmade and perceived nutritional quality (H3).

### 7.1 Theoretical implications

First, the primary contribution of this study lies in uncovering the positive influence of handmade on consumers’ perceived nutritional quality of organic food, thereby offering a novel perspective for the marketing of organic food. Our findings demonstrate that handmade not only enhances consumers’ perceived nutritional quality of organic food (20), but also strengthen their trust and satisfaction toward the corresponding products. In line with the existing research (52), our study not only confirmed that handmade food can better attract consumers’ purchase intentions, but emphasized that handmade organic food can more effectively stimulate consumers’ perceived nutritional quality of organic food. Additionally, these discoveries hold significant implications for organic food manufacturers and marketers, which allow they to highlight the importance of conveying manufacturers’ intentions and integrity, through product details and quality optimization, which can effectively embellish consumers’ overall perception of organic food (53). Consequently, manufacturers can enhance product quality through handmade production, while marketers can augment consumers’ trust and satisfaction, by emphasizing the value of handmade products, thereby fostering product sales and market acceptance (54).

Second, the study found that handicraft cultural identity serves as a mediating factor between handmade and consumers’ perceived nutritional quality of organic food, which is crucial for the development of the organic food market. As people’s awareness of health and environmental protection has been increased, the demand for organic food continues to grow, but the lack of consumer cognition poses a challenge for marketing (55). Therefore, understanding consumers’ perception of handmade food is essential for the production and marketing of organic food. Our research advanced the existing study (56), further demonstrated that cultural identity not only influences consumers’ purchase intentions but also helps consumers enhance their perception of product quality. This study revealed that handicraft cultural identity mediates the relationship between handmade organic food and perceived nutritional quality, indicating that consumers tend to perceive handmade organic food as healthier and more nutritious, because it better meets consumer expectations. These findings provide a clear direction for organic food manufacturers to improve consumers’ perceived nutritional quality of organic food by enhancing product quality and developing handicraft cultural identity.

Last, the study found that perceived authenticity moderated the relationship between handmade and consumers’ perceived nutritional quality, which is particularly important for the organic food market. With rising health awareness, consumer demand for organic food is growing, and they are increasingly concerned about the original and nutritional quality of food. Organic food manufacturers and marketers need to have a deep understanding of this consumers’ psychology, in order to improve the attractiveness and market competitiveness of their products (55). Research has shown that by improving the perceived authenticity of their products, organic food manufacturers can attract consumers and enhance product competitiveness (57). For instance, consumers’ perceptions of food safety and nutritional quality can be enhanced through organic certification and the provision of detailed nutritional information (58). In addition, marketing strategies should also highlight the safety and nutritional benefits of products to attract consumers. At the same time, understanding consumers’ concerns about the authenticity and nutritional quality of organic food can help manufacturers and marketers better meet consumer needs and increase their satisfaction and recognition of certain products. Manufacturers and marketers can emphasize these characteristics to attract consumers’ attention and motivate their purchase decision-making.

### 7.2 Managerial implications

First, this study revealed that handmade enhances the quality and taste of organic food, such as the fluffiness of handmade bread and the fineness of cheese. By carefully controlling the production process, manufacturers not only enhance the attractiveness of their products, but also increase consumers’ awareness and trust. Improved technology and awareness of quality are essential to meet market demand and are key to increasing consumer confidence and driving market growth (59). The organic food market is receiving more attention with rising health and environmental awareness, but there is also intense competition. We suggest that marketers should make full use of the advantages of handmade to strengthen consumer trust and satisfaction, as a strategy to increase sales and market share.

Second, handmade products are effective in enhancing consumers’ cultural identity with organic food due to their uniqueness and individuality. At a time when consumers generally have reservations about industrial production, handmade products are perceived as more delicate and mindful, therefore more reliable and trustworthy (60). We believe that product differentiation is the key to attracting consumers in a competitive market. Handmade products are able to differentiate themselves from other products due to their uniqueness, and provide a distinctive consumer experience. Organic food marketers can create product differentiation to attract consumers’ attention and motivate their purchase decision-making, by emphasizing the unique value of handmade.

Last, with the rise of health awareness, organic food has been favored by more and more consumers due to its natural and pollution-free properties (61). Although the nutritional value of organic food has been widely recognized (62), the lack of awareness among many consumers affects positive consumer behavior. This study investigated the effect of handmade on consumers’ perceived nutritional quality of organic food and found that handmade significantly increased consumers’ recognition and trust in the nature and nutritional quality of organic food. This finding has important implications for stimulating consumers’ health awareness and promoting their consumption behavior. Handmade not only deepens consumers’ understanding of the health value of organic food, but also enhances their trust in the quality of the product, which makes them more willing to purchase and consume organic food. Therefore, we suggest that handmade should be used to promote the popularity and development of organic food, by encouraging consumers to participate more actively in the production and consumption of organic food.

### 7.3 Limitations and future research

However, this study needs to acknowledge certain limitations. For example, the sample size may limit the generalizability of the findings, the experimental conditions may affect the accuracy of the results, and the impact of cultural differences on consumers’ perceptions has not been adequately considered. In addition, the study mainly focused on short-term effects, with insufficient understanding of the impact on long-term behavior, and further validation is needed since consumer preferences and behavioral patterns may change with changes in the market environment. Future research could extend to consumers from different cultural backgrounds, conduct long-term tracking studies, analyze the impact of handmade in a multi-dimensional way, and explore the possibility of combining modern technology with traditional handmade. At the same time, research on how to raise consumers’ awareness of the value of organic food and handmade products through education and publicity, constructing models of consumer behavior and conducting market segmentation, as well as research on the impact of handmade products on the environment and how to achieve sustainable development, are all important future directions.

## Data Availability

The raw data supporting the conclusions of this article will be made available by the authors, without undue reservation.

## Footnotes

^1^https://www.credamo.com/
